# Does teaching qualification matter in higher education in the UK? An analysis of National Student Survey data

**DOI:** 10.1016/j.mex.2019.04.001

**Published:** 2019-04-06

**Authors:** Mohammad Nurunnabi, Abdelhakim Abdelhadi, Rehab Aburas, Samaher Fallatah

**Affiliations:** a Prince Sultan University, P.O. Box 66833, Riyadh, 11586, Saudi Arabia; b St Antony’s College, University of Oxford, 62 Woodstock Road, Oxford, OX2 6JF, UK

**Keywords:** Survey Questionnaire, Teaching Qualification Data, Higher Education Academy (HEA), Student satisfaction, National Student Survey (NSS), Russell Group, United Kingdom

## Abstract

This article investigates the relationship between the teaching quality and student satisfaction in higher education institutions in the United Kingdom (UK). 121 universities were randomly selected for this data article. The findings reveal that a higher percentage of Higher Education Academy (HEA) qualification among universities’ staff is positively associated with higher ratings of student satisfaction. Non-Russell Group universities have a greater proportion of HEA qualified teachers than do Russell Group universities. Of the 10 highest-ranking universities for student satisfaction, only two are Russell Group Universities (Newcastle University and University of Oxford). The findings may inform policy implications.

• Little research has focused on the relationship between teaching qualification and student satisfaction. This article adds value by identifying an association between teaching qualifications and student satisfaction in UK higher education institutions.
• The validity of the data was assured through its collection from various sources, including a survey questionnaire from the National Student Survey (NSS), The Russell Group (UK), The Higher Education Academy (HEA), and Higher Education Funding Council for England (Hefce), UK.
• The data can be used by the scientific community to understand the prevalence of teaching qualification in higher education, and factors associated with student satisfaction in the UK. The data can also be useful for higher education policymakers in the UK. The data could be applied to exploring differences in student perceptions of teaching quality (e.g. between local and international students, or students of different genders).

## Graphical abstract

**Figure:**
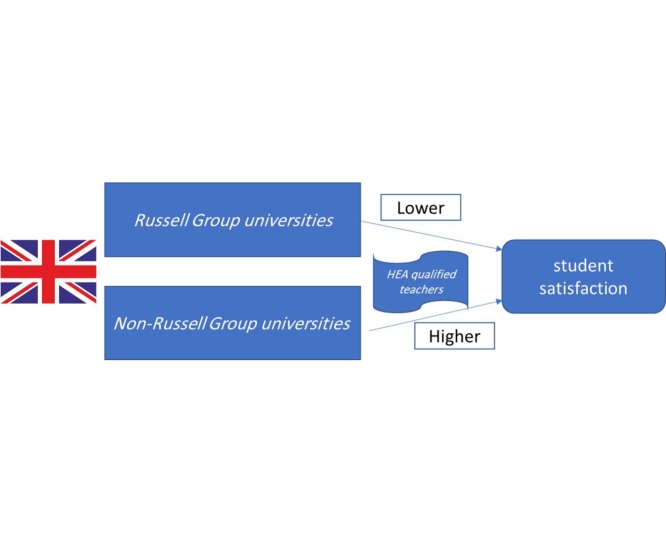

**Specifications Table**

**Table:** 

**Subject Area:**	*Social Sciences*
**More specific subject area:**	*Higher Education*
**Method name:**	*Survey Questionnaire; Teaching Qualification Data*
**Name and reference of original method**	*Higher Education Funding Council for England, National Student Survey, (2015), Available at:*http://www.hefce.ac.uk/lt/nss/*(accessed 12 November 2017).*
**Resource availability**	*The data is available in the article*

## Method details

Student evaluation has become a key metric by which administrators evaluate the teaching quality of the faculty they oversee [[1], [2], [3], [4]]. A higher rating on student satisfaction is positively correlated with the students’ grades in those courses [[5], [6], [7], [8], [9], [10], [11], [12], [13]]. The data were collected from various sources: National Student Survey (NSS), the Higher Education Academy (HEA), and the Russell Group (UK). The Russell Group comprises 24 world-class, research-intensive universities. The member universities of the Russell Group have significant social, economic and cultural impacts nationally and globally. For example, most of the world-class research is produced by the Russell Group universities in the UK and their economic output is more than £32 billion per year. According to the Russell Group, “They are unique institutions, each with their own history and ethos, but they share some distinguishing characteristics. They are committed to maintaining the very best research, an outstanding teaching and learning experience and unrivalled links with local and national business and the public sector” (https://russellgroup.ac.uk/).

### National Student Survey (NSS)

The National Student Survey (NSS) in the UK gathers students’ opinions on the quality of their courses to ensure public accountability. Because this survey is based on student experience, the results (published on the Unistats website – https://unistats.ac.uk) inform prospective students, thereby assisting them in selecting institutions to attend. The NSS respondents are mainly final-year undergraduates studying for higher education qualifications at UK higher education providers and at further education colleges in England, Northern Ireland and Wales. The NSS includes the following areas:

A Teaching and learning
B Assessment and feedback
C Academic support
D Organization and management
E Learning resources
F Personal development
G Overall satisfaction

To date, the NSS has helped over two million students make their voices heard and has helped to bring about significant and positive change in higher education in the UK. In 2018, the NSS results cover the views of 320,000 students [14]. The overall satisfaction in 2018 is 83%, compared with 84% in 2017 [14].

### The Higher Education Academy (HEA)

The Higher Education Academy (HEA) is the national body in the UK for championing teaching excellence and wider student learning experience. The HEA works with governments, ministries, universities and individual academics in the UK and around the globe. The members of the HEA are Universities UK (UUK) and GuildHE (https://www.heacademy.ac.uk/).

As a teaching qualification in higher education, the HEA Fellowship demonstrates a personal and institutional commitment to professionalism in learning and teaching. As of 2018, there are around 108,000 fellows of the HEA. The following four categories of fellowships recognize the practice, impact and leadership of individuals’ teaching and learning:

• Associate Fellowship (AFHEA)
• Fellowship (FHEA)
• Senior Fellowship (SFHEA)
• Principal Fellowship (PFHEA).

The Fellowship is awarded based on evidence of personal professional practice that meets the requirements of one of the four Descriptors of the UK Professional Standards Framework (UKPSF) (https://www.heacademy.ac.uk/ukpsf).

### Sample

Table 1 shows the sample of the study (n = 121). The study includes 19 Russell Group and 102 Non-Russell Group universities. Student satisfaction ranges from 74% to 95% in higher education institutions in UK. The percentage of faculty with HEA qualification in universities ranges from 0% to 94%. The percentage of faculty with total teaching qualification (including HEA qualification and other teaching qualifications) ranges from 3% to 90%. The number of staff in the universities ranges from 25 to 4065.

**Table 1:** Teaching Qualification of the Sample institution (n = 121).

Institution	Russel Group	Teaching qualification	HEA Qualification	Student Satisfaction
Imperial College London	*Russell Group*	23%	52%	88%
King's College London	*Russell Group*	41%	26%	81%
Queen Mary University of London	*Russell Group*	33%	34%	88%
The London School of Economics and Political Science	*Russell Group*	13%	39%	81%
The University of Birmingham	*Russell Group*	45%	47%	88%
The University of Leeds	*Russell Group*	40%	38%	90%
The University of Liverpool	*Russell Group*	34%	41%	85%
The University of Manchester	*Russell Group*	68%	28%	86%
The University of Nottingham	*Russell Group*	49%	54%	86%
The University of Sheffield	*Russell Group*	37%	59%	90%
The University of Warwick	*Russell Group*	37%	29%	87%
University College London	*Russell Group*	23%	18%	83%
University of Bristol	*Russell Group*	31%	24%	84%
University of Cambridge	*Russell Group*	3%	44%	90%
Durham University	*Russell Group*	59%	33%	90%
University of Exeter	*Russell Group*	51%	69%	90%
Newcastle University	*Russell Group*	40%	62%	91%
University of Oxford	*Russell Group*	17%	30%	91%
University of York	*Russell Group*	41%	31%	88%
Anglia Ruskin University	*Non-Russell Group*	72%	47%	85%
Aston University	*Non-Russell Group*	60%	56%	90%
Bath Spa University	*Non-Russell Group*	40%	32%	90%
Birkbeck College	*Non-Russell Group*	41%	48%	87%
Birmingham City University	*Non-Russell Group*	44%	39%	81%
Bishop Grosseteste University	*Non-Russell Group*	75%	44%	85%
Bournemouth University	*Non-Russell Group*	58%	35%	79%
Brunel University London	*Non-Russell Group*	41%	61%	85%
Buckinghamshire New University	*Non-Russell Group*	60%	27%	82%
Canterbury Christ Church University	*Non-Russell Group*	68%	28%	87%
City, University of London	*Non-Russell Group*	26%	37%	87%
Courtauld Institute of Art	*Non-Russell Group*	13%	0%	94%
Coventry University	*Non-Russell Group*	42%	28%	91%
De Montfort University	*Non-Russell Group*	39%	32%	86%
Edge Hill University	*Non-Russell Group*	79%	19%	85%
Falmouth University	*Non-Russell Group*	41%	31%	84%
Goldsmiths' College	*Non-Russell Group*	9%	20%	83%
Guildhall School of Music & Drama	*Non-Russell Group*	19%	31%	83%
Harper Adams University	*Non-Russell Group*	78%	80%	93%
Heythrop College	*Non-Russell Group*	43%	26%	90%
Kingston University	*Non-Russell Group*	59%	34%	82%
Leeds Beckett University	*Non-Russell Group*	55%	43%	82%
Leeds College of Art	*Non-Russell Group*	66%	11%	81%
Leeds Trinity University	*Non-Russell Group*	72%	30%	85%
Liverpool Hope University	*Non-Russell Group*	70%	49%	89%
Liverpool John Moores University	*Non-Russell Group*	55%	53%	85%
London Metropolitan University	*Non-Russell Group*	65%	36%	79%
London South Bank University	*Non-Russell Group*	44%	35%	82%
Loughborough University	*Non-Russell Group*	42%	40%	91%
Manchester Metropolitan University	*Non-Russell Group*	35%	38%	85%
Middlesex University	*Non-Russell Group*	69%	21%	83%
Newman University	*Non-Russell Group*	68%	36%	89%
Norwich University of the Arts	*Non-Russell Group*	44%	34%	87%
Nottingham Trent University	*Non-Russell Group*	56%	35%	88%
Oxford Brookes University	*Non-Russell Group*	43%	30%	90%
Plymouth College of Art	*Non-Russell Group*	74%	2%	74%
Ravensbourne	*Non-Russell Group*	32%	21%	80%
Roehampton University	*Non-Russell Group*	68%	67%	83%
Rose Bruford College of Theatre and Performance	*Non-Russell Group*	49%	38%	89%
Royal College of Music	*Non-Russell Group*	6%	0%	86%
Royal Holloway, University of London	*Non-Russell Group*	38%	33%	89%
Royal Northern College of Music	*Non-Russell Group*	29%	16%	86%
Sheffield Hallam University	*Non-Russell Group*	55%	38%	85%
Southampton Solent University	*Non-Russell Group*	63%	37%	82%
St Mary's University, Twickenham	*Non-Russell Group*	37%	31%	88%
St. George's, University of London	*Non-Russell Group*	58%	35%	86%
Staffordshire University	*Non-Russell Group*	48%	40%	83%
Teesside University	*Non-Russell Group*	84%	24%	86%
The Arts University Bournemouth	*Non-Russell Group*	37%	36%	81%
The Conservatoire for Dance and Drama	*Non-Russell Group*	30%	8%	91%
The Liverpool Institute for Performing Arts	*Non-Russell Group*	63%	75%	88%
The Open University	*Non-Russell Group*	46%	23%	90%
The Royal Academy of Music	*Non-Russell Group*	10%	10%	82%
The Royal Agricultural University	*Non-Russell Group*	73%	47%	89%
The Royal Central School of Speech and Drama	*Non-Russell Group*	36%	43%	77%
The Royal Veterinary College	*Non-Russell Group*	60%	14%	92%
The School of Oriental and African Studies	*Non-Russell Group*	3%	44%	87%
The University of Bath	*Non-Russell Group*	35%	44%	90%
The University of Bolton	*Non-Russell Group*	66%	28%	83%
The University of Bradford	*Non-Russell Group*	62%	36%	85%
The University of Chichester	*Non-Russell Group*	63%	43%	88%
The University of Cumbria	*Non-Russell Group*	76%	16%	78%
The University of East Anglia	*Non-Russell Group*	38%	35%	92%
The University of Essex	*Non-Russell Group*	42%	35%	92%
The University of Huddersfield	*Non-Russell Group*	90%	37%	88%
The University of Hull	*Non-Russell Group*	52%	94%	86%
The University of Kent	*Non-Russell Group*	29%	34%	89%
The University of Lancaster	*Non-Russell Group*	33%	22%	91%
The University of Leicester	*Non-Russell Group*	44%	27%	85%
The University of Northampton	*Non-Russell Group*	34%	17%	86%
The University of Reading	*Non-Russell Group*	46%	30%	89%
The University of Salford	*Non-Russell Group*	29%	42%	83%
The University of Surrey	*Non-Russell Group*	53%	39%	92%
The University of West London	*Non-Russell Group*	59%	16%	79%
The University of Westminster	*Non-Russell Group*	49%	27%	80%
The University of Wolverhampton	*Non-Russell Group*	71%	37%	82%
Trinity Laban Conservatoire of Music and Dance	*Non-Russell Group*	24%	33%	80%
University College Birmingham	*Non-Russell Group*	78%	54%	82%
University for the Creative Arts	*Non-Russell Group*	49%	28%	81%
University of Bedfordshire	*Non-Russell Group*	43%	50%	83%
University of Brighton	*Non-Russell Group*	45%	83%	83%
University of Central Lancashire	*Non-Russell Group*	49%	33%	85%
University of Chester	*Non-Russell Group*	82%	69%	88%
University of Derby	*Non-Russell Group*	67%	44%	88%
University of East London	*Non-Russell Group*	37%	43%	78%
University of Gloucestershire	*Non-Russell Group*	55%	55%	83%
University of Greenwich	*Non-Russell Group*	51%	30%	83%
University of Hertfordshire	*Non-Russell Group*	53%	41%	84%
University of Keele	*Non-Russell Group*	61%	32%	95%
University of Lincoln	*Non-Russell Group*	52%	32%	85%
University of Northumbria at Newcastle	*Non-Russell Group*	61%	39%	88%
University of Plymouth	*Non-Russell Group*	73%	21%	87%
University of Portsmouth	*Non-Russell Group*	40%	34%	89%
University of St Mark & St John	*Non-Russell Group*	79%	84%	78%
University of Sunderland	*Non-Russell Group*	53%	35%	86%
University of Sussex	*Non-Russell Group*	32%	24%	87%
University of the Arts, London	*Non-Russell Group*	15%	40%	75%
University of the West of England, Bristol	*Non-Russell Group*	54%	34%	85%
University of Winchester	*Non-Russell Group*	45%	28%	92%
University of Worcester	*Non-Russell Group*	66%	29%	87%
Writtle University College	*Non-Russell Group*	69%	24%	75%
York St John University	*Non-Russell Group*	83%	75%	88%

**Fig. 1:**
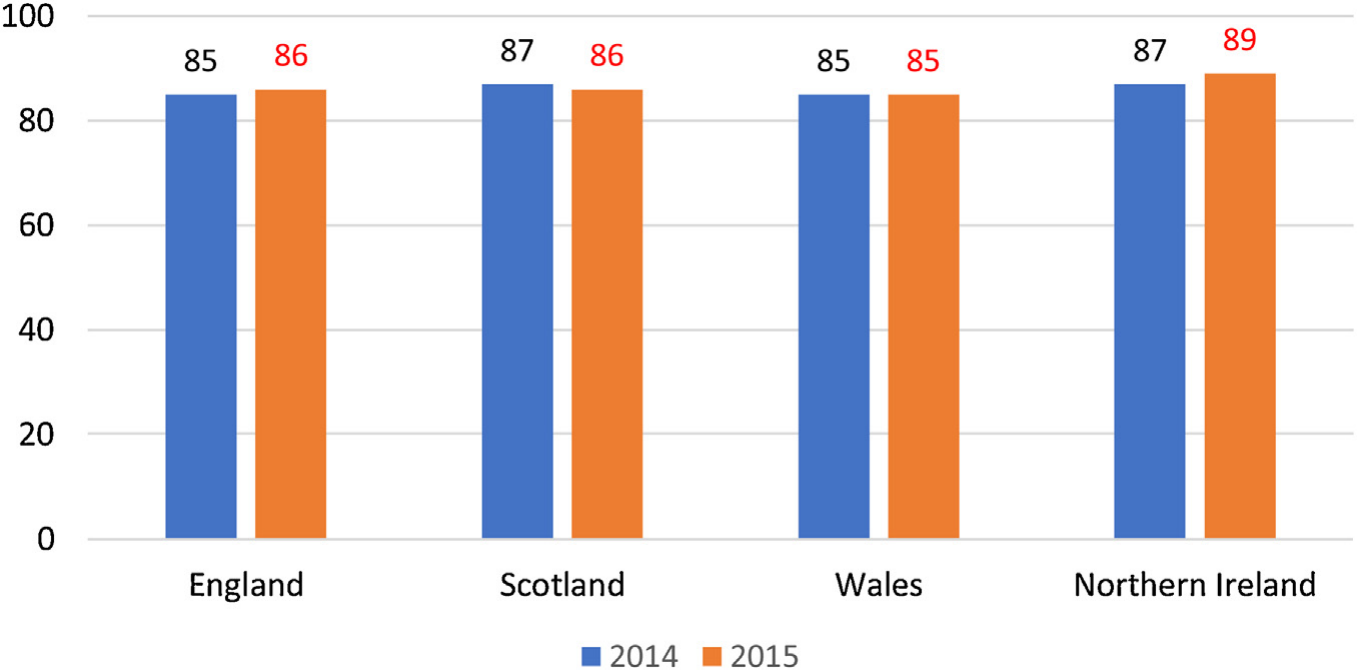
Full-time Student satisfaction percentage in four regions in UK.

**Fig. 2:**
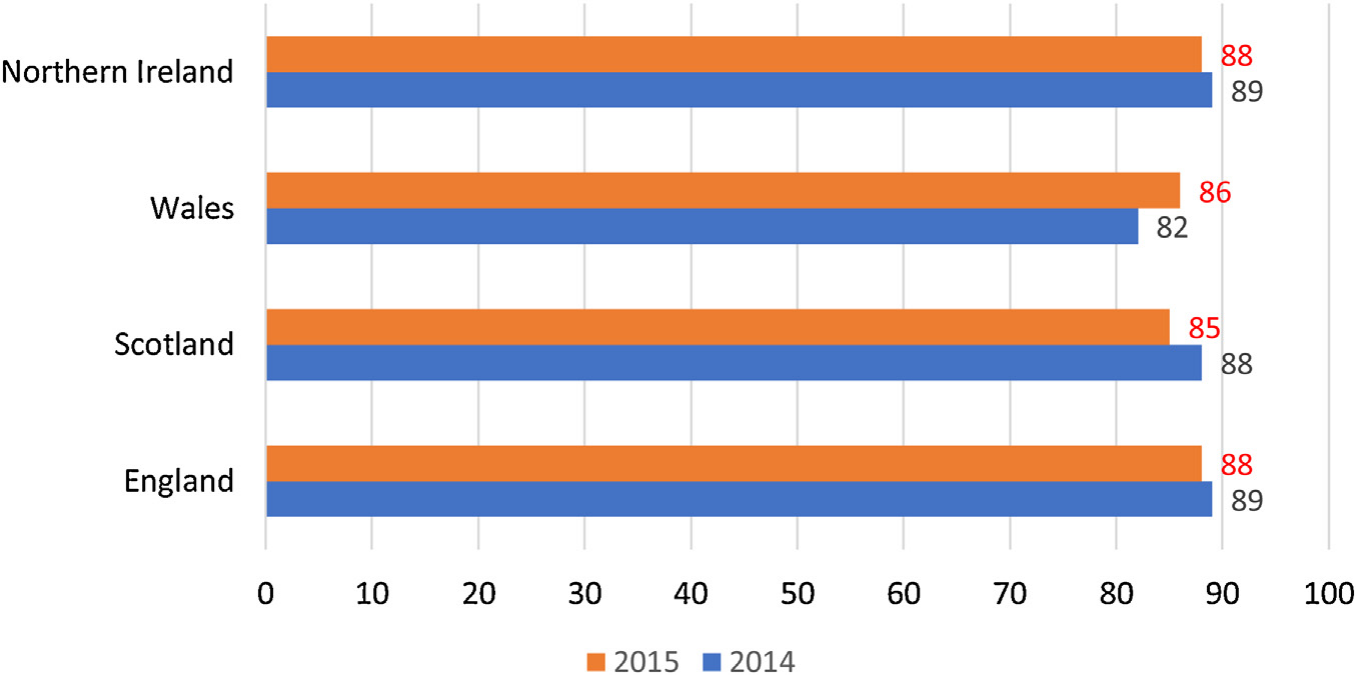
Part-time Student satisfaction percentage in four regions in UK.

**Fig. 3:**
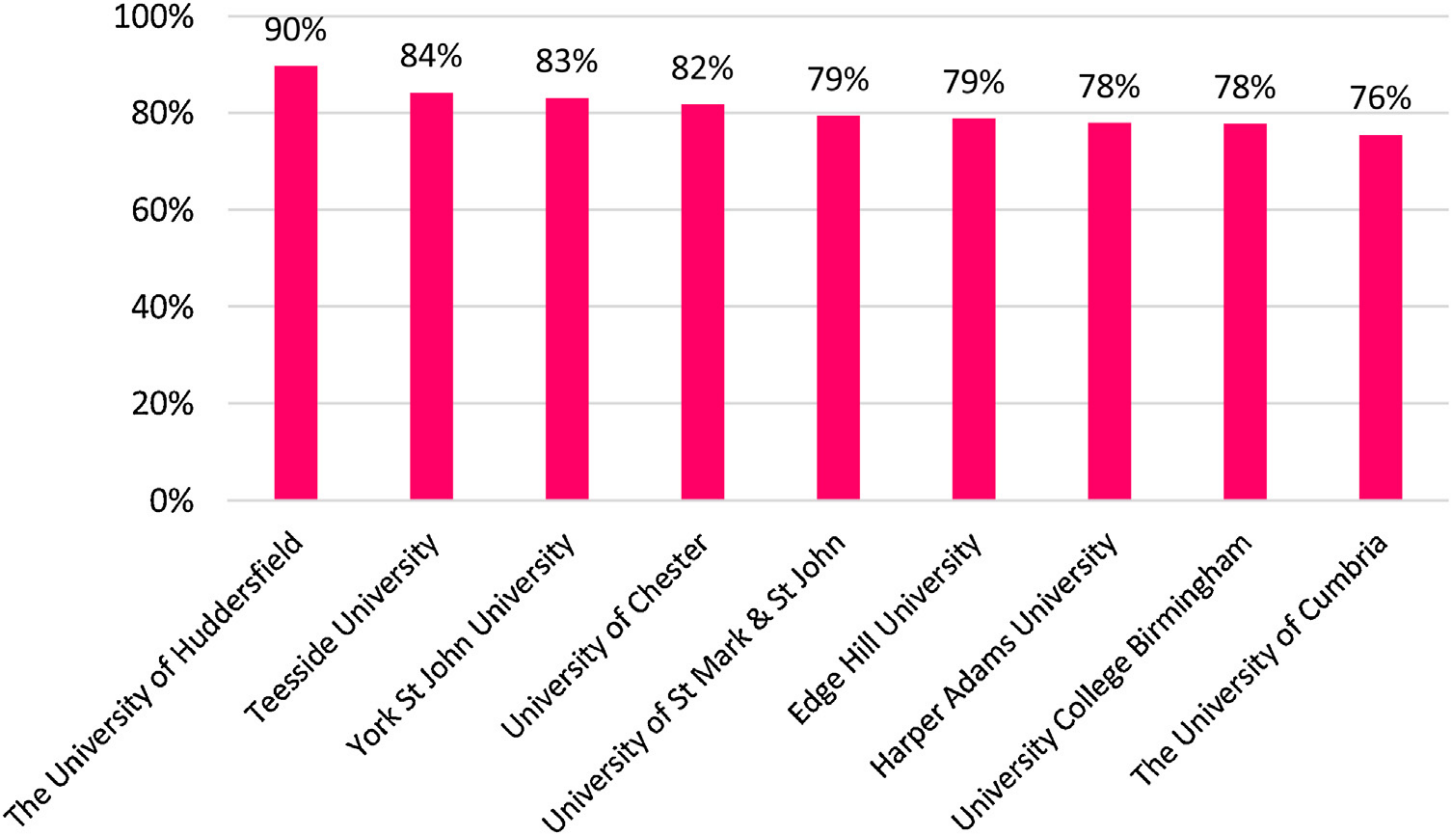
Teaching qualification held (Highest percentage).

**Fig. 4:**
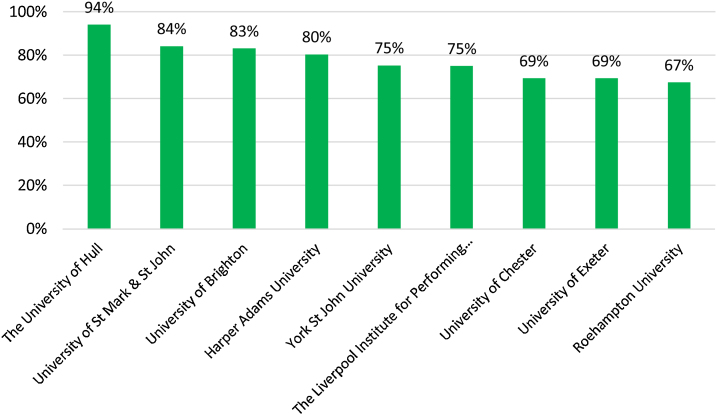
HEA Qualification (Highest percentage).

The full-time student satisfaction percentage in 2015 was higher for the three regions (England, Scotland and Northern Ireland) than Wales (see Fig. 1). Fig. 2 presents that the part-time student satisfaction percentage in 2015 was the lowest in Scotland (85%) in 2015. Fig. 3 shows that all of the nine universities with the highest percentages of teachers having total teaching qualifications were Non-Russell Group universities: The University of Huddersfield, Teesside University, York St John University, University of Chester, University of St Mark & St John, Edge Hill University, Harper Adams University, University College Birmingham, and The University of Cumbria. Fig. 4 shows that the highest percentages of teaching staff with HEA Qualification were predominantly (with the exception of University of Exeter) in Non-Russell Group universities: The University of Hull, University of St Mark & St John, University of Brighton, Harper Adams University, York St John University, The Liverpool Institute for Performing Arts, University of Chester, and Roehampton University.

## Experimental design

To achieve the objective of the data article, the study has developed the following hypothesis under three models (Russell Group and Non-Russell Group):

**Model I:**

$$\begin{array}{cc} \text{Null}\;\text{hypothesis}: & H_{0}:\mu_{1}-\mu_{2}=0 \end{array}$$

$$\begin{array}{cc} \text{Alternative}\;\text{hypothesis}: & H_{1}:\mu_{1}-\mu_{2}\neq 0 \end{array}$$

Where, *μ*_1_: mean of Teaching qualification held; *μ*_2_: mean of Teaching qualification held, R (Russell-Group); Difference: *μ*_1_ − *μ*_2_

**Model II:**

$$\begin{array}{cc} \text{Null}\;\text{hypothesis}: & H_{0}:\mu_{1}-\mu_{2}=0 \end{array}$$

$$\begin{array}{cc} \text{Alternative}\;\text{hypothesis}: & H_{1}:\mu_{1}-\mu_{2}\neq 0 \end{array}$$

Where, *μ*_1_: mean of R-Overseas accreditation or qualification (Russell-Group); *μ*_2_: mean of Overseas accreditation or qualification; Difference: *μ*_1_ − *μ*_2_

**Model III:**

$$\begin{array}{cc} \text{Null}\;\text{hypothesis}: & H_{0}:\mu_{1}-\mu_{2}=0 \end{array}$$

$$\begin{array}{cc} \text{Alternative}\;\text{hypothesis}: & H_{1}:\mu_{1}-\mu_{2}\neq 0 \end{array}$$

Where, *μ*_1_: mean of Other UK accreditation or qualification; *μ*_2_: mean of R Other UK accreditation or qualification; Difference: *μ*_1_ − *μ*_2_

As presented in Table 2, Model I shows that the mean proportion of staff holding a Teaching Qualification is 0.357 for Russell Group universities and 0.505 for Non-Russell Group universities. Model II shows that the mean proportion of staff holding a Teaching Qualification (Overseas accreditation or qualification for any level of teaching) is 0.079 for Russell Group universities and 0.037 for Non-Russell Group universities. Model III shows that the mean proportion of staff holding a Teaching Qualification (Other UK accreditation or qualification in teaching in higher education) is 0.076 for Russell Group universities and 0.047 for Non-Russell Group universities. The standard deviation of Model I is higher than Model II and Model III.

**Table 2:** Descriptive Statistics.

	Sample	N	Mean	SD	SE Mean
Model I	Teaching Qualification held, R (Russell Group)	19	0.357	0.166	0.040
	Teaching Qualification held (Non-Russell Group)	102	0.505	0.186	0.018
Model II	Teaching Qualification (Overseas accreditation or qualification for any level of teaching), R (Russell Group)	19	0.079	0.053	0.013
	Teaching Qualification (Overseas accreditation or qualification for any level of teaching) (Non-Russell Group)	102	0.037	0.045	0.004
Model III	R – Teaching Qualification (Other UK accreditation or qualification in teaching in higher education) (Russell Group)	19	0.076	0.057	0.014
	Teaching Qualification (Other UK accreditation or qualification in teaching in higher education) (Non-Russell Group)	102	0.047	0.038	0.003

Regarding the Estimation for Difference (see Table 3), the confidence interval at 95% is: −0.056 ≤ μ_1_ − μ_2_ ≤ 0.239 (Model I), 0.012 ≤ μ_1_ − μ_2_ ≤ 0.072 (Model II), and −0.060 ≤ μ_1_ − μ_2_ ≤ 0.003 (Model III).

**Table 3:** Estimation for Difference.

Model	Difference	95% CI for Difference
Model I	0.1477	(0.056, 0.239)
Model II	0.0424	(0.012, 0.072)
Model III	−0.0289	(−0.060, 0.003)

**Fig. 5:**
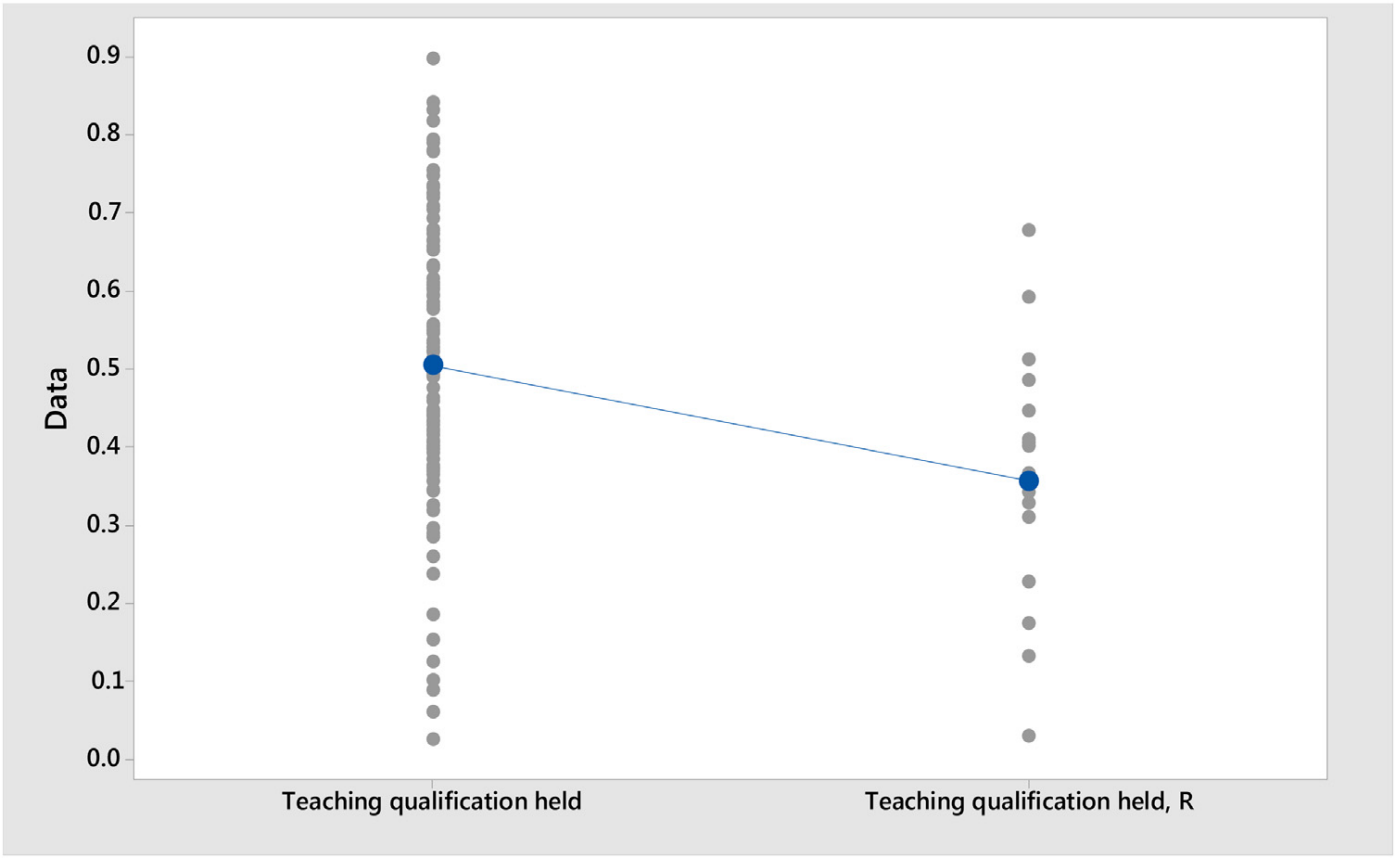
Individual Value Plot of Teaching Qualification. *Note*: Teaching Qualification held, R = Russell Group; Teaching Qualification held = Non-Russell Group.

**Fig. 6:**
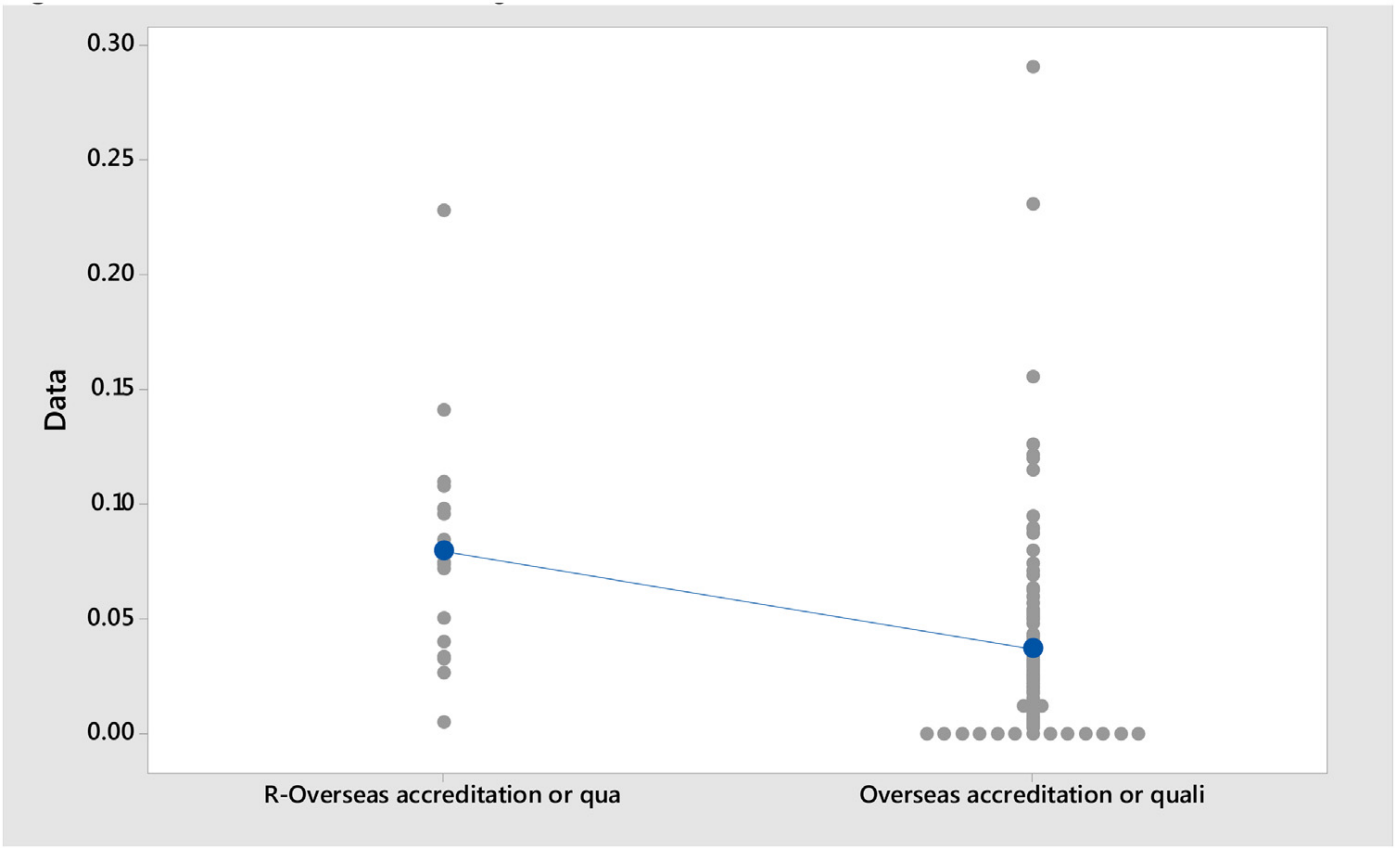
Individual Value Plot of Teaching Qualification. *Note*: R – Teaching Qualification (Overseas accreditation or qualification for any level of teaching), R = Russell Group; Teaching Qualification = Non-Russell Group.

**Fig. 7:**
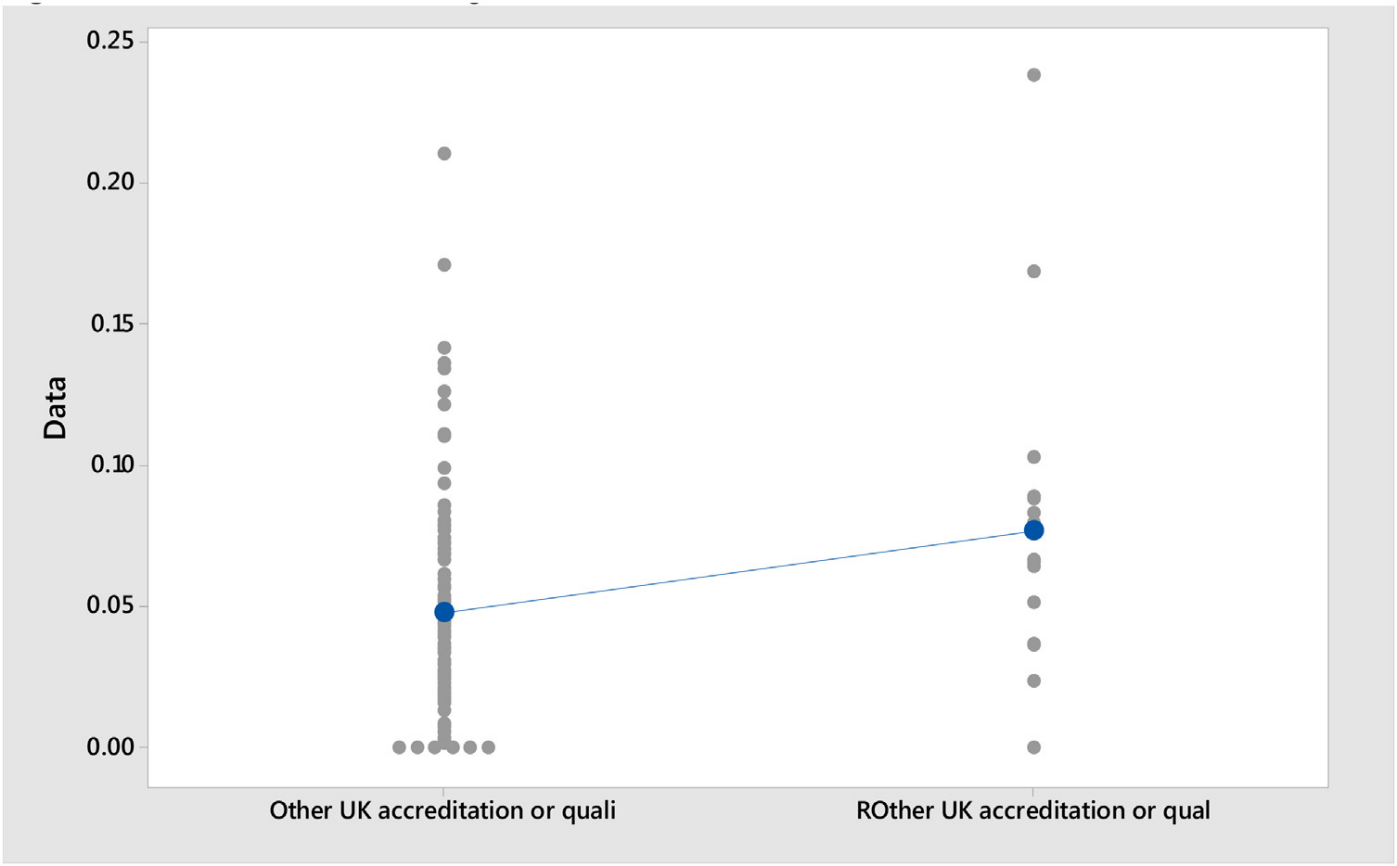
Individual Value Plot of Teaching Qualification. *Note*: R – Teaching Qualification (Other UK accreditation or qualification in teaching in higher education) = Russell Group; Teaching Qualification (Other UK accreditation or qualification in teaching in higher education) = Non-Russell Group.

Table 4 presents the results of the *t*-test. *T*-Test is applied to measure the difference of teaching Qualification held between Russell Group and Non-Russell Group Universities. Model I contain t value is 3.34, df is 23 and p < .05 (p = 0.003), and therefore the null hypothesis is rejected. Model II contain t value is 2.99, df is 18 and p < .05 (p = 0.008), and therefore the null hypothesis is rejected. Model III contain t value is −1.94, df is 17 and p > .05 (p = 0.069), and therefore the null hypothesis cannot be rejected. Fig. 5, Fig. 6, Fig. 7 show the individual Value Plots for Russell Group and Non-Russell Group. The results of the *t*-test show that overall Teaching Qualification (Overseas accreditation or qualification for any level of teaching) are significantly different between Russell Group and Non-Russell Group. But Teaching Qualification (Other UK accreditation or qualification in teaching in higher education) for Russell Group and Non-Russell Group are not significantly different.

**Table 4:** *t*-Test.

Model	*t*	*df*	*p*
Model I	3.34	23	0.003
Model II	2.99	18	0.008
Model III	−1.94	17	0.069

**Fig. 8:**
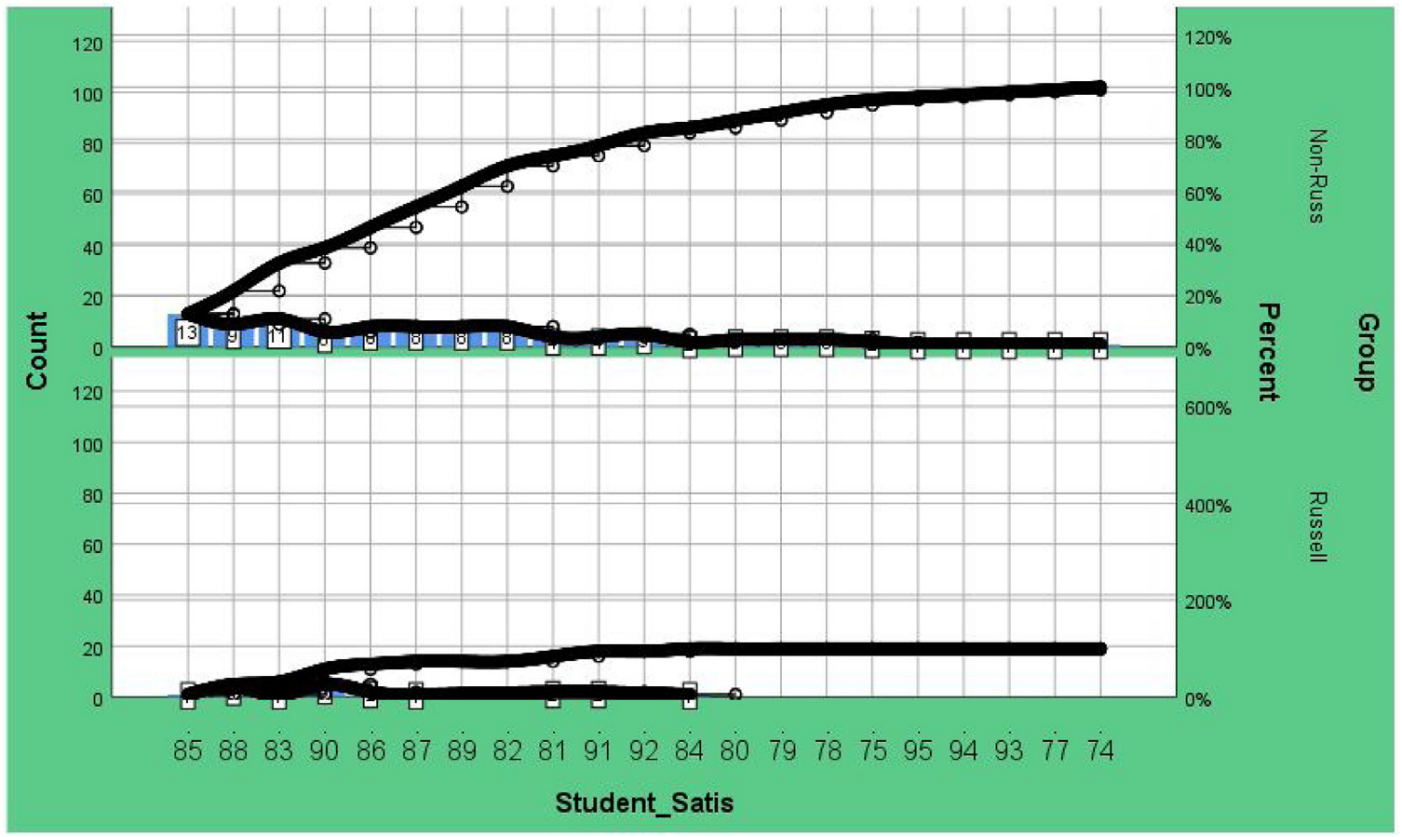
Student satisfaction of Russell Group and Non-Russell Group.

**Fig. 9:**
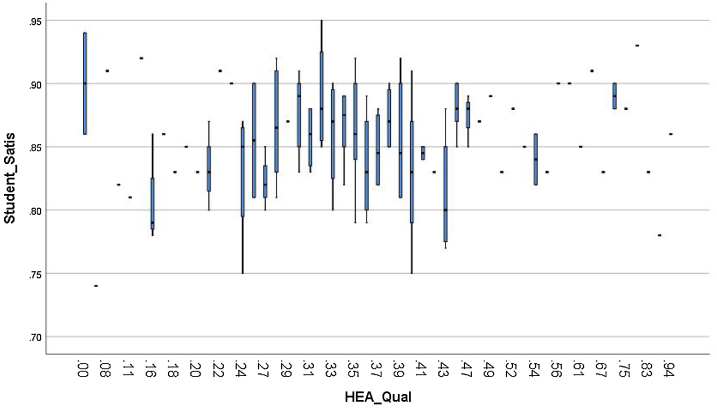
Student satisfaction and HEA Teaching Qualification.

Regarding the comparison between teaching qualification and student satisfaction, Fig. 8 presents the Student satisfaction for Russell Group and Non-Russell Group universities. The mean Student satisfaction for Russell Group is 87%, while for Non-Russell Group it is 85.48%. Fig. 9 shows that HEA Teaching qualification is associated with higher student satisfaction.

As presented in Table 5, Pearson Correlation shows that there was a positive correlation between HEA Qualification and Teaching Qualification, which was statistically significant (r = 0.242, n = 121, p = .008). This means that most of the qualified teachers were HEA qualified.

**Table 5:** Pearson Correlation.

Variables	Student_Satis	HEA_Qual	Teaching_Qual	Total_staff
Student_Satis	1			
HEA_Qual	0.090	1		
Teaching_Qual	−0.084	0.242**	1	
Total_staff	0.125	−0.135	0.029	1

*Note*: Dependent variable: Student_Satis; Independent variable: HEA_Qual, Teaching_Qual andTotal_staff.

** Correlation is significant at the 0.01 level (2-tailed).

Finally, Table 6 shows the results of regression analysis for students’ satisfaction. It is evident from the results that model fits the data well (p < .05) and there is a strong positive relationship between dependent variables and independent variables. The independent variables of the model explain 34% of the variations in the dependent variable. The variables when compared on individual basis, only faculties with HEA qualification variable is significant (p < .05). This reveals that faculties with HEA qualification in universities is positively associated with student satisfaction.

**Table 6:** Regression result.

Variables	Unstandardized Coefficients		Standardized Coefficients	t	Sig.	Collinearity Statistics	
	B	Std. Error	Beta			Tolerance	VIF
(Constant)	0.841	0.014		59.407	0.000		
HEA_Qual	0.045	0.024	0.173	1.849	**0.067**	0.949	1.054
Teaching_Qual	−0.020	0.021	−0.089	−0.962	0.338	0.965	1.036
Total_staff	1.051E-05	0.000	0.151	1.641	0.103	0.979	1.021

*Note*: Dependent variable: Student_Satis; Independent variable: HEA_Qual, Teaching_Qual andTotal_staff ; p value = significance value. The bold value indicates p < .05.

## Conflict of interest

The authors declare no conflict of interest.
